# Quantifying the complexity of black-and-white images

**DOI:** 10.1371/journal.pone.0207879

**Published:** 2018-11-26

**Authors:** Damián H. Zanette

**Affiliations:** Centro Atómico Bariloche and Instituto Balseiro, Comisión Nacional de Energía Atómica and Universidad Nacional de Cuyo, Consejo Nacional de Investigaciones Científicas y Técnicas, 8400 San Carlos de Bariloche, Río Negro, Argentina; Consejo Nacional de Investigaciones Cientificas y Tecnicas, ARGENTINA

## Abstract

We propose a complexity measure for black-and-white (B/W) digital images, based on the detection of typical length scales in the depicted motifs. Complexity is associated with diversity in those length scales. In this sense, the proposed measure penalizes images where typical scales are limited to small lengths, of a few pixels –as in an image where gray levels are distributed at random– or to lengths similar to the image size –as when gray levels are ordered into a simple, broad pattern. We introduce a complexity index which captures the structural richness of images with a wide range of typical scales, and compare several images with each other on the basis of this index. Since the index provides an objective quantification of image complexity, it could be used as the counterpart of subjective visual complexity in experimental perception research. As an application of the complexity index, we build a “complexity map” for South-American topography, by analyzing a large B/W image that represents terrain elevation data in the continent. Results show that the complexity index is able to clearly reveal regions with intricate topographical features such as river drainage networks and fjord-like coasts. Although, for the sake of concreteness, our complexity measure is introduced for B/W images, the definition can be straightforwardly extended to any object that admits a mathematical representation as a function of one or more variables. Thus, the quantification of structural richness can be adapted to time signals and distributions of various kinds.

## Introduction

“Bacteria, for example, are probably *no more complex* than their ancestors 2000 million years ago. The most that we can say is that some lineages have become *more complex* in the course of time. Complexity is hard to define or to measure but there is surely some sense in which elephants and oak trees are *more complex* than bacteria, and bacteria than the first replicating molecules.” (John Maynard Smith and Eörs Szathmáry, 1995 [1]).

“The term ‘mark’ derives from its earliest use in phonology. The marked member is *relatively complex* in relation to the unmarked. Thus in our previously cited example, the nasal vowel is *more complex* acoustically than its oral counterpart in that it involves nasal resonances in addition to the oral resonances of oral vowels.” (Joseph Greenberg, 1980 [2]).

“…as was already mentioned, attractors in dissipative chaos are generally not points, not lines, but are *more complex*, and their complex geometrical nature is expressed by the fact that they are fractals.” (Ilya Prigogine, 1990 [3]).

Although coming from three disparate areas of scientific knowledge, all of the above texts –authored by very influential scholars of the last few decades, with emphases added by us– implicitly address the possibility of comparing complexity between entities of a given class: organisms, in the case of Maynard Smith and Szathmáry; phonemes, in the case of Greenberg; and geometrical objects, in the case of Prigogine. A prerequisite for comparing complexity of different objects, in turn, is to possess a way to assert how complex an object is. In the scientific literature, complexity measures began to be systematically addressed during the 1980s, when the theory of complex systems was acquiring sufficient consistency as to become a fruitful background for interdisciplinary research [4].

A plausible measure of complexity is given by the information needed to specify the structure, the state, or the evolution of the object under study. This notion is particularly adapted to systems for which an entropy can be defined, as in the case of probability measures, symbolic sequences, and a large class of physical systems, among others. In fact, entropy is a straightforward statistical quantification of the difficulty of ascertaining the specific features being described [5]. Information and entropy, in turn, are closely related to the concepts of algorithmic (Kolmogorov) complexity [6] and data compressibility [7], which are routinely used in computer science to gauge the intricacy of problems in fields such as communication, information flow, and decision making [8].

By definition, however, entropy quantifies disorder. As a consequence, any complexity measure purely based on entropy estimations will generally assign higher values to features with larger proportions of random ingredients. This direct connection between complexity and randomness is unsatisfactory in many contexts [9]. Within statistical approaches, specifically, the analysis often becomes less involved when the degree of randomness grows. For instance, the statistical-mechanical description of a gas is much simpler than that of a liquid, despite the fact that molecular motions are less disordered in the latter than in the former. In such cases, randomness corresponds to lower complexity. From a broader perspective, thus, complexity measures are expected to capture the subtle balance between order and disorder –flexibility and organization, diversity and systematicity– that distinguishes, for instance, living beings from inanimate matter [10]. This point of view was first put forward by Weaver in 1948 [11], and extensively discussed in quantitative terms from the 1980s. It was early adopted to measure complexity in computational algorithms, from an information-theoretical perspective [12], as well as in thermodynamic states of physical systems, where the notions of order and disorder admit a well-defined quantification [13, 14]. Similar approaches were soon applied to all kinds of natural and artificial objects, ranging from dynamical and out-of-equilibrium systems [15, 16], to hierarchical structures [17], cellular automata and Boolean networks [18], glassy systems [19], and many others.

In this contribution, we propose a complexity measure which, in principle, could be applied to any entity that admits a mathematical representation as a function defined over a bounded domain of arbitrary dimensionality. This wide class spans such disparate objects as a time series obtained from an experimental measurement, the instantaneous distribution of particles in a volume of gas, or the terrain elevation in a given region of the Earth. For the sake of concreteness, however, this complexity measure is not introduced *in abstracto*, but with reference to a specific application, namely, quantifying the complexity of black-and-white (B/W) digital images. In fact, B/W images can be conceived as a distribution of gray levels over a two-dimensional –typically, rectangular– domain, and therefore provide an opportune illustration of the present proposal. Much along the ideas outlined in the previous paragraph, our complexity measure aims to quantify the distance to both order and disorder. In the case of B/W images, these two extremes can be respectively conceived as the pictorial representation of a simple pattern, with typical lengths of the order of the image size, and a completely random distribution of grays, where the only detectable structures are occasional small-scale regularities caused by local aleatory fluctuations. Complexity, in contrast, is expected to take its largest values for images with a wealth of structural details, at many length scales.

It is worth mentioning that quantifying image complexity is a crucial step in the exploration of high-level cognitive functions related to human visual perception, such as aesthetic appraisal [20, 21]. Discerning between objective and subjective complexity is necessary for the correct assessment of the stimulus-response connection up to the highest stages of our brain’s visual pathway [20, 22]. The choice of B/W images to illustrate our complexity measure, therefore, is also motivated by its possible applications to visual perception research.

Our approach is based on an automated procedure that, analyzing a B/W image at different resolution levels, is able to identify the typical length scales of the depicted motifs. These structural scales are then integrated into a single index that quantifies complexity, by direct comparison with the extreme cases of fully ordered and disordered images. Several images are compared with each other on the basis of this complexity index, an application to the analysis of complexity in topographic data is presented, and a variety of aspects and possible extensions of the approach are discussed.

## Results

### Detecting typical scales in a black-and-white digital image

For the present purposes, a black-and-white (B/W) digital image is conceived as a rectangular array of pixels, where each pixel is characterized by its gray level. Assuming the standard gray-level resolution of 8 bits per pixel, the gray level of each pixel is an integer number which varies between 0 and 255. Our quantification of complexity is based on the analysis of local variations of the gray level for a series of versions of the same image at successively decreasing spatial resolutions. Details are given in the Methods section. For each spatial resolution, we measure the variance of the gray level inside small domains at different points on the image, and then average the obtained variances over the whole image. The resulting *mean variance* *V* is assigned to a *scale* *S*, given in pixels, which depends on the resolution and on the size of the domains where the variance is computed. The scale *S* defines the typical length, measured on the original image, over which the gray-level variance is being calculated. As the resolution is changed, the scale varies, and a value of the mean variance for each scale, *V* (*S*), is determined.

The above described procedure is a variant of a method proposed in the frame of the so-called *scale-space theory* [23] to detect characteristic scales in an image. According to this formulation, for an image portraying a pattern with a well defined typical length, the gray-level mean variance *V* (*S*) attains a maximum at a scale which is directly related to that length [24]. In the Methods section we outline a mathematical proof of this fact. Semiquantitatively, it is clear that, for scales much smaller than the typical length of image patterns, the mean variance will be small. In turn, for values of *S* much larger than the typical lengths, successive resolution reductions would have smoothed out the image structure, and *V* (*S*) will again be small. Higher values of the mean variance are expected for intermediate scales, around the typical length of the depicted patterns.

**Fig 1:**
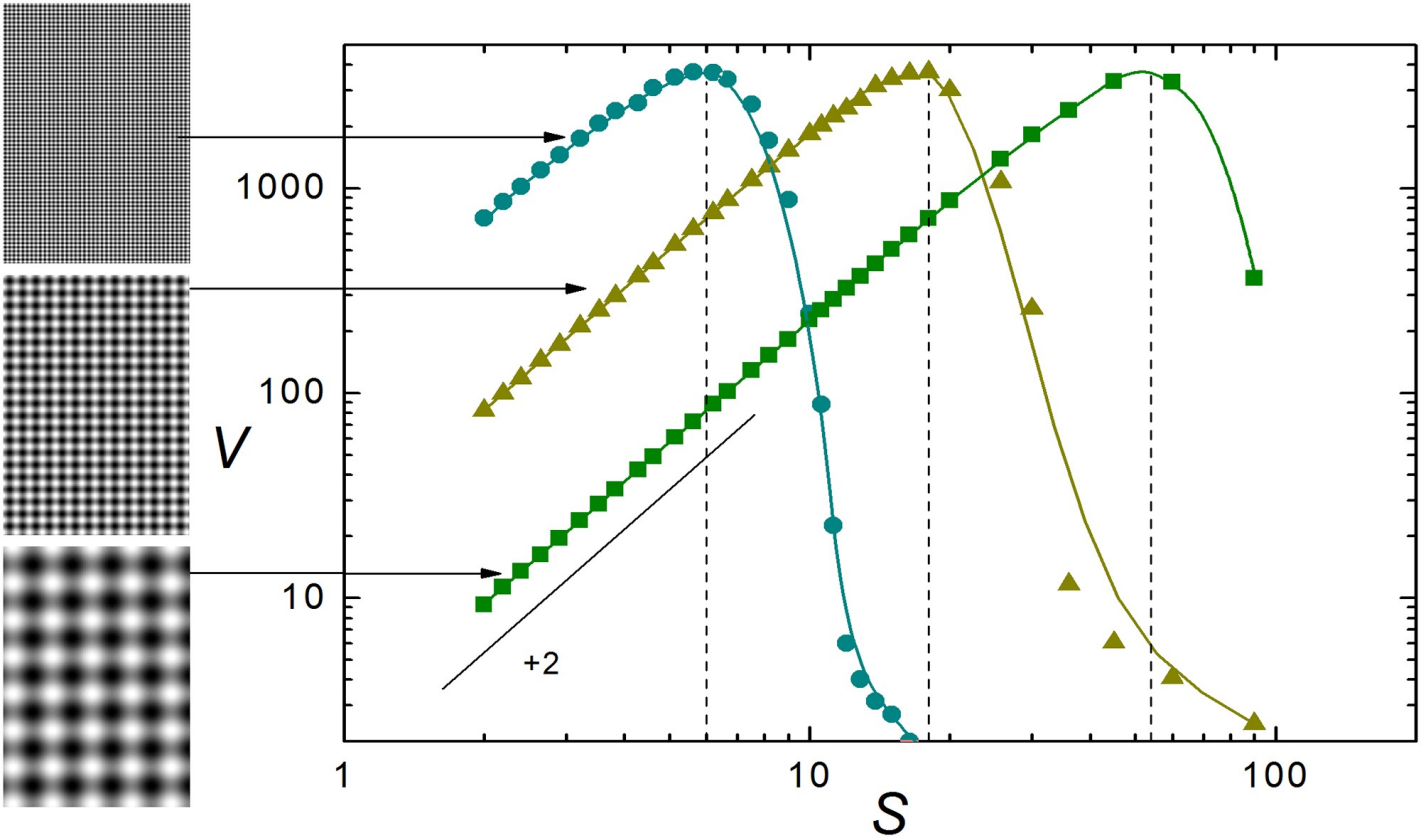
The gray-level mean variance *V* as a function of the scale *S*, measured in pixels, for the three 360 × 540 images shown to the left (circles: Upper image; triangles: Middle image; squares: Lower image). Curves are B-spline approximations for log *V* as a function of log *S*. Vertical dashed lines stand at the values of *S* coinciding with the side of the square cells in each image. The slanted segment has slope +2, representing the algebraic functional relation *V* (*S*) ∝ *S*^2^ in the log-log plot. The B/W images in this figure are the author’s work. They are available as S1 File.

As an illustration, Fig 1 shows the gray-level mean variance *V* as a function of the scale *S*, measured in pixels, for the three images shown in the leftmost column. The images, which are all 360 × 540 pixels in size (aspect ratio 2: 3), portray smoothed chessboard-like patterns, with different offsets with respect to the edges. From top to bottom, the side of the square cells in each image are 6, 18, and 54 pixels. In the plot, dots stand for the computed mean variances, and curves are B-spline interpolations included for clarity. In the three cases, *V* (*S*) attains its maximum at a value of *S* which closely coincides with the side of the cells, as shown by the vertical dashed lines. These examples demonstrate that, as predicted by scale-space theory, the position of the maxima provides an excellent estimate of the typical length scale of the patterns depicted in the images. This prediction will here be used as the basic hypothesis to detect structure in complex images.

### Images with several typical scales

**Fig 2:**
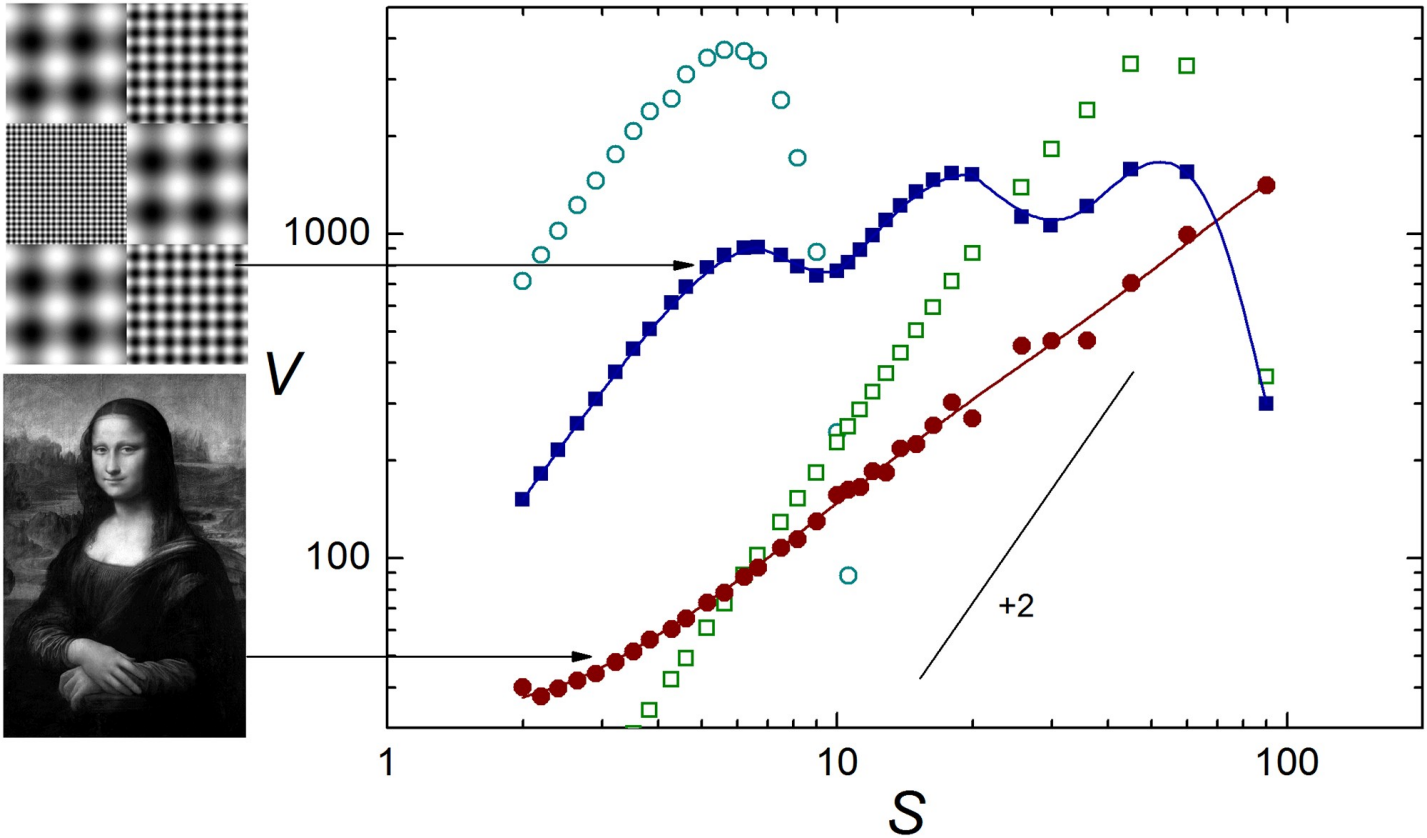
As in Fig 1, for the two 360 × 540 images shown to the left (full squares: Upper image; full circles: Lower image). Empty symbols correspond to the same data shown in Fig 1, for the uppermost and lowermost images in its left column. The B/W images in this figure are either the author’s work or copyright-free material in the public domain, downloaded from http://commons.wikimedia.org. They are available as S1 File.

Take now the two 360 × 540 images in the leftmost column of Fig 2. The upper one is a mosaic combining six 180 × 180 patches taken from the three images of Fig 1. Full squares in Fig 2 stand for its mean variance. For comparison, the plot also includes the data already shown in Fig 1 for the chessboard-like images with cells of size 6 pixels (empty circles) and 54 pixels (empty squares). For the new image, the mean variance exhibits three local maxima, at positions coinciding with the maxima for the images of Fig 1. Clearly, *V* (*S*) is capturing the combination of scales present in the new image and, at the same time, displays a much broader shape.

The other image in Fig 2 is Leonardo da Vinci’s Mona Lisa, a portrait painted at the beginning of the sixteenth century. The corresponding mean variance is shown in the plot as full circles. Overall, its profile is much smoother than for the upper image. On the average, moreover, the slope is substantially lower than for the chessboard-like images. According to the above interpretation for the upper image, *V* (*S*) must be incorporating contributions of typical lengths at various scales *S*. Inspection of Mona Lisa’s portrait, taking into account details both in the main subject (face, hair, dress) and in the richly structured background landscape, qualitatively supports the assertion that many typical lengths are adding to *V* (*S*). Indeed, in the 360 × 540 resolution of the original image, pictorial elements span scales from a few pixels to large portions of the canvas. As advanced in the Introduction, it is this structural richness what we aim to associate with complexity in an image. A criterion to quantify image complexity may therefore arise from a measure of the flatness of *V* (*S*): contributions coming from many scales should tend to produce a more horizontal profile for the gray-level mean variance.

To further explore this idea, and in order to perform a more consistent comparison between different images, let us analyze a set of images that not only coincide in size –as in the cases shown in Figs 1 and 2– but which are formed by exactly the same collection of pixels, i.e. with coincident distributions of grays. Different profiles for *V* (*S*) can thus be purely attributed to the specific way in which the same pixels are positioned over each image [21]. In the following, the Mona Lisa image of Fig 2 is taken as the source of the pixel collection.

**Fig 3:**
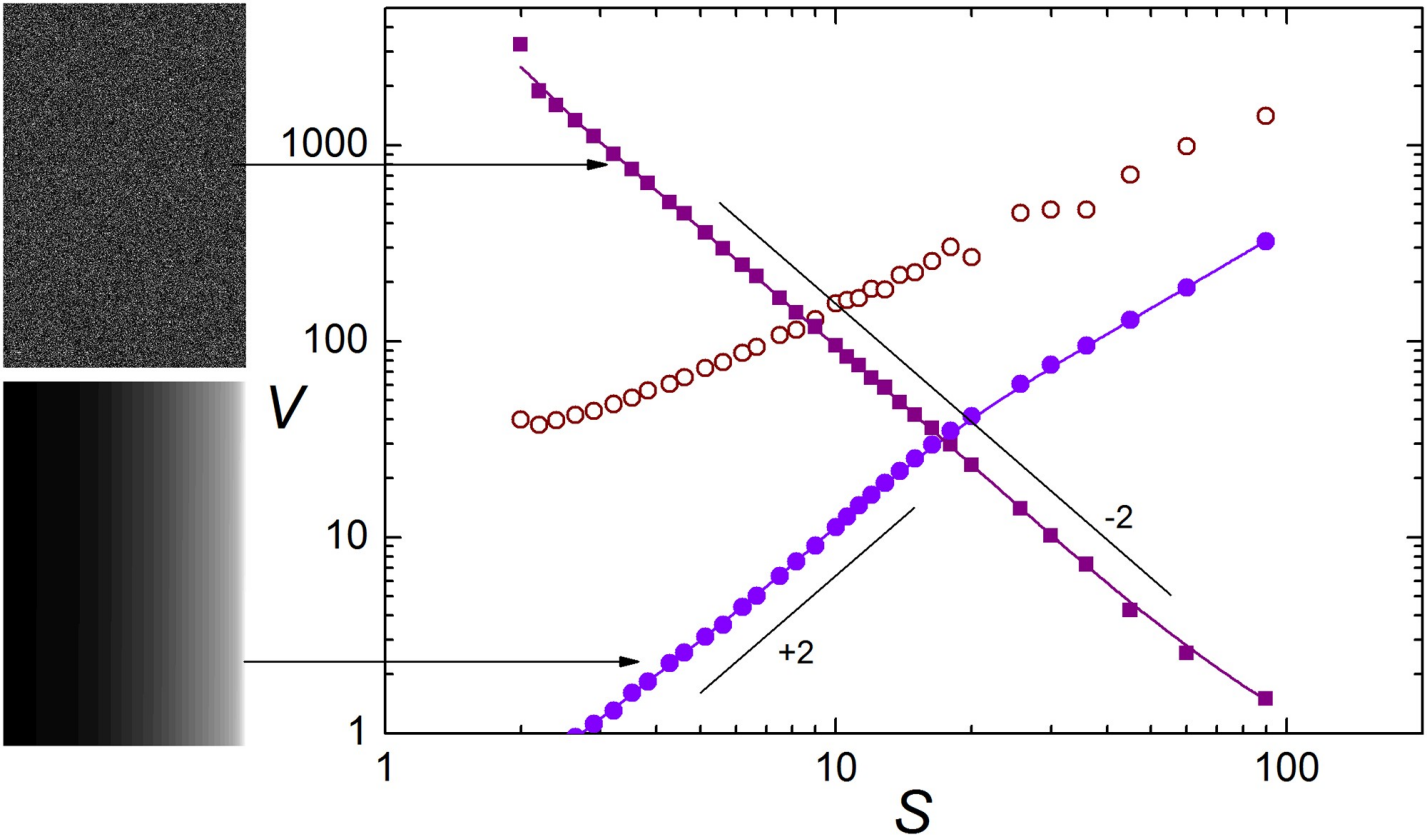
As in Fig 1, for the two 360 × 540 images shown to the left, both obtained as rearrangements of the pixels in the Mona Lisa image of Fig 2 (full squares: Upper image—Random rearrangement; full circles: Lower image—Ordered rearrangement). Empty circles correspond to the Mona Lisa data, also shown in Fig 2. The slanted segments have slope −2 and +2, respectively representing the algebraic functional relations *V* (*S*) ∝ *S*^−2^ and *V* (*S*) ∝ *S*^2^ in the log-log plot. The B/W images in this figure are the author’s work. They are available as S1 File.

Consider first two rearrangements of the Mona Lisa pixels whose complexity –according to the concepts discussed in the Introduction– should reach the lowest values. One is an image where pixels are redistributed at random. In the other image, pixels are orderly arranged by their brightness from the upper-left corner (darkest gray) to the lower-right corner (brightest gray). Both are shown in the left column of Fig 3. In the random image, apart from local fluctuations, no discernible structure is present. In the ordered image, the only apparent structure is the ramp from darker to lighter grays, spanning the whole image width. The main panel shows *V* (*S*) for the two images (full symbols), and for Mona Lisa (empty circles). We see that the variance of the random image closely follows an algebraic decay of exponent −2, namely, *V* (*S*) ∝ *S*^−2^. For the ordered image, the dependence is also approximately algebraic, *V* (*S*) ∝ *S*^*γ*^, with a positive exponent very close to 2 for *S* ≲ 20 pixels, and a lower exponent (*γ* ≈ 1.4) for larger scales. Both profiles are significantly steeper, in either direction, than for the Mona Lisa image.

As a matter of fact, it is possible to give a simple analytical argument showing that the variance of a random image should depend on the scale as *V* (*S*) ∝ *S*^−2^. Similarly, under rather general conditions, the ordered gray distribution should have a variance close to *V* (*S*) ∝ *S*^2^. These arguments are outlined in the Methods section. Note that the functional relation *V* (*S*) ∝ *S*^2^ is also present in the data shown in Fig 1 for the small-*S* range. At the shortest scales, in fact, the images considered in Fig 1 and the ordered rearrangement of Mona Lisa are statistically very similar, with smoothly varying shades of gray.

Thus, for a given collection of pixels, the slopes −2 and 2 in the log-log plot of *V* (*S*) turn out to be a compact characterization of the functional relations between mean variance and scale for the extreme cases of fully random and fully ordered images, which we aim to associate with the lowest complexity values. This suggests that a quantification of complexity may result from a suitably defined distance between a given variance profile and the functional relations characterized by the two slopes, assigning larger complexity values to flatter profiles. Using this idea, in the Methods section we define an index *Q* which quantifies the degree of complexity of a B/W image, and thus makes it possible to compare images with each other.

**Fig 4:**
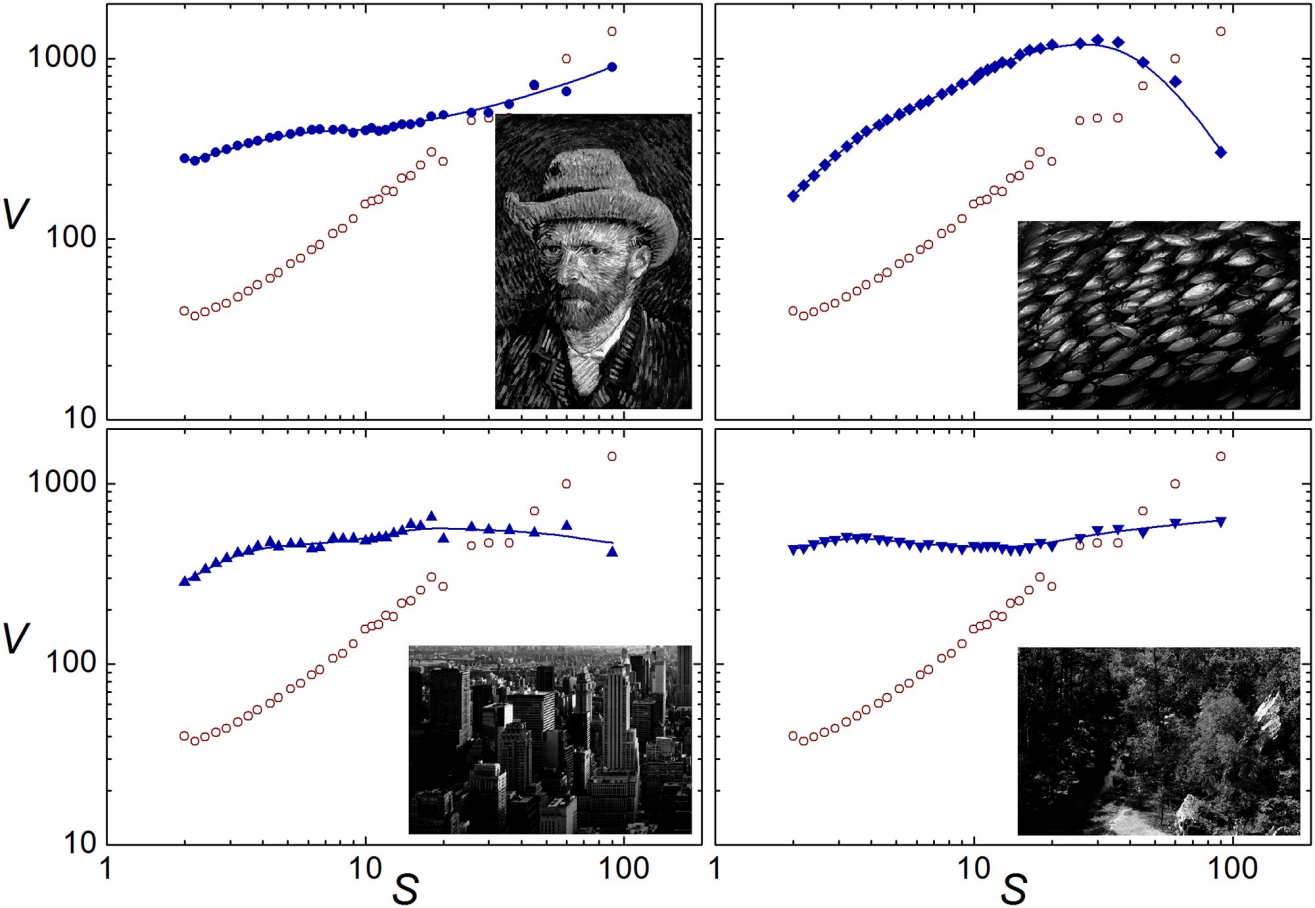
As in Fig 1, for four rearrangements of the Mona Lisa pixels. In each panel, full and empty symbols respectively correspond to the image shown in the inset and to the 360 × 540 Mona Lisa image. Upper-left: Vincent van Gogh’s “Self-Portrait with Grey Felt Hat”. Upper-right: A photograph of a fish school. Lower-left: Urban landscape in New York city. Lower-right: A forest landscape. The B/W images in this figure are either the author’s work or copyright-free material in the public domain, downloaded from http://commons.wikimedia.org. They are available as S1 File.

Before reporting the results of computing the complexity index *Q*, let us explore how the gray-level mean variance behaves for a few other rearrangements of the Mona Lisa pixels. To generate these rearrangements, we choose a reference image of the same size as Mona Lisa, and use it as a “mold” to redistribute the pixels, as follows. We rank the pixels in the reference image and in Mona Lisa by their gray level –for instance, from brightest to darkest. The relative ranking of pixels with identical gray level in each image is irrelevant to the procedure. Then, we replace each pixel in the reference image by the pixel with the same rank in Mona Lisa. The resulting image is a rearrangement of the Mona Lisa pixels with the same semblance as the reference image (see the examples in Fig 4).

The upper-left panel of Fig 4 shows the result of the variance analysis for a rearrangement of the Mona Lisa pixels using as a reference image a self-portrait by Vincent van Gogh, painted in the 1880’s. Full symbols in the plot represent *V* (*S*) for the van Gogh image (shown in the inset), while empty circles correspond to the original Mona Lisa image, already shown in Figs 2 and 3. Comparing with Mona Lisa, the substantially larger values of *V* (*S*) for small *S* (≲ 20 pixels) in the van Gogh portrait are to be ascribed to the clearly visible paintbrush strokes, characteristic of the Impressionist artist’s style, which add a rich small-scale structure to the image. In sharp contrast, Mona Lisa is a paramount example of Leonardo’s *sfumato* style, where the purposely blended adjacent surfaces make the brushstrokes indiscernible. The abundant small-scale details of the van Gogh image thus result in a flatter profile for *V* (*S*).

The other examples shown in Fig 4 take as reference images three photographs of the real world. In the three cases, the aspect ratio is rotated 90° with respect to Mona Lisa but, within our methodology, this does not affect the comparison. The upper-right image shows a fish school. Since the salient feature in this photograph is the repeated shape of individual fish, not unexpectedly, *V* (*S*) shows a maximum at *S* ≈ 25 pixels, which broadly coincides with the typical length of a fish on the image. The lower-left image portrays an urban landscape which, due to perspective effects, exhibits details of many sizes. The resulting profile for *V* (*S*) is, as in the case of van Gogh’s self-portrait, significantly flatter than for Mona Lisa. An even flatter profile is obtained for the lower-right photograph, a forest landscape dominated by foliage of varied brightness. The wealth of details at all scales –in particular, in the distribution of light and shadows– determines that the mean variance is virtually independent of scale.

Table 1 shows the complexity index *Q* obtained for the Mona Lisa image and all the rearrangements considered in Figs 3 and 4, ordered by increasing values of *Q*. By definition (see Methods), the numerical value of the complexity index is restricted to the interval [0, 1]. Larger values correspond to images with flatter variance profiles. Overall, the resulting ordering of these images seems to agree with an intuitive notion of complexity which captures structural richness at different scales.

**Table 1:** Complexity index for various B/W images.

Image	Complexity index *Q*
Random	0.063
Ordered	0.283
Fish school	0.754
Mona Lisa	0.764
van Gogh’s self-portrait	0.971
Urban landscape	0.978
Forest landscape	0.991

The complexity index *Q* for the Mona Lisa image of Fig 2, and all the rearrangements considered in Figs 3 and 4, ordered by increasing values of *Q*.

### Application: A complexity map for South-American topography

As an application of the quantification of complexity in B/W digital images, we have studied the distribution of the complexity index *Q* over a large image representing the topography of South America. As a source, we used the “Topography” digital mosaic from NASA’s Visible Earth (Blue Marble) collection –a gray-scale representation of elevation data obtained by space-based radars all over our planet [25]. We combined the two 10800 × 10800 tiles which cover the South-American continent, cropped the combination using a 2: 3 aspect ratio frame, and rescaled the resulting image to a final size of 2560 × 3540 pixels. The 2560 × 3540 image can be divided into 40 × 60 = 2400 non-overlapping sub-images of 64 × 64 pixels. For each one of these sub-images, we have calculated the index *Q*, following the same procedure as above. Moreover, to test the consistency of the results, we have repeated the analysis with 32 × 32 sub-images (9600 in number), obtaining –up to variations associated with the different size of the sub-images– a fully compatible distribution for the complexity index.

**Fig 5:**
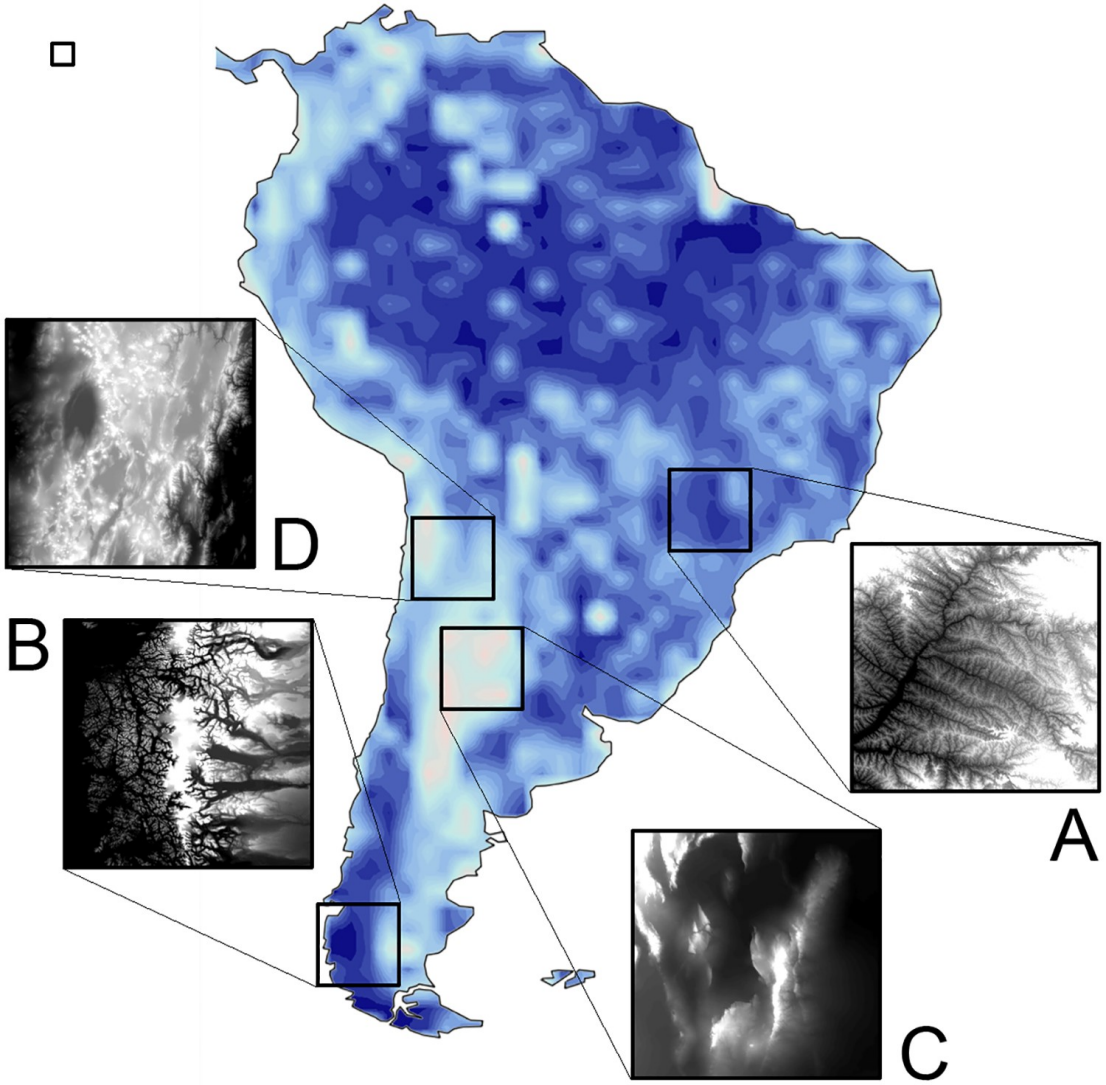
Central panel: A complexity map of the South-American topography. Darker and brighter shades respectively correspond to larger and lower values of the complexity index *Q*. The insets A to D display selected sectors, 256 × 256 pixels in size, of the original B/W image, which codes terrain elevations as gray levels. The small square in the upper-left corner shows the relative size of the 64 × 64 sub-images used to determine *Q* all over the original image. The original B/W image is copyright-free material (https://visibleearth.nasa.gov/useterms.php) from NASA Visible Earth collection, downloaded from https://visibleearth.nasa.gov/view.php?id=73934 [25]. The cropped South-America image is available as S1 File.

The central panel of Fig 5 shows a contour plot of the distribution of the index *Q* over the topography image of South America, constructed from the grid of results obtained for the 2400 sub-images. Darker and brighter shades respectively stand for larger and lower values of *Q*. The small square frame in the upper-left corner shows the relative size of each 64 × 64 sub-image. The four larger frames scattered over the plot correspond to 256 × 256 sectors. The inset connected to each frame shows the respective sector of the original image, with its contrast enhanced for better visibility.

At first sight, it may come as a surprise that the largest zone with high values of *Q* spreads out over the Amazonian forest –a very flat region, mainly within Brazil, in the upper-central part of the map– while, except at the southern end, *Q* is comparatively small on the mountainous Andean area, along the western coast. We must bear in mind, however, that the index *Q* is designed to detect multiplicity in structural scales, rather than just variability in the gray levels. To elucidate this point, it is useful to inspect some selected spots of the *Q* distribution and the corresponding sectors of the original image.

Inset A shows a 256 × 256 sector in south-central Brazil, where *Q* attains a relatively large value as compared with the surroundings. The spot turns out to coincide with the uppermost basin of Paraná River, the second longest in South America. The intricate network of a multitude of tributaries, converging to increasingly larger streams, translates into a wealth of details at many scales. Indeed, river drainage networks are one of the best-known examples of real-world fractals [26]. The self-similar nature of these geomorphological features, whose forms copy each other at different typical lengths, is consistent with a large value of *Q*. Note that the large-*Q* broad arc surrounding the Amazonian region covers the upper drainage basin of the other two large South-American rivers, Amazon and Orinoco.

Another spot of large *Q* occurs on the southernmost Pacific coast in Chile (inset B). The extremely labyrinthine coastline, formed by myriads of fjords, has also been carved by a drainage system –in this case, of glaciers from the Southern Patagonian Icefields– and shares the self-similar geometry of river networks. Coastlines, in fact, are another classical example of natural fractals [27]. In the zone shown by inset B, drainage occurs also eastward, into the Patagonian steppe, where the complexity index decreases rapidly in the direction of the Atlantic Ocean.

At the other extreme, the largest region of low-*Q* values covers most of central Argentina. Inset C shows that this zone is not free of topographical features. In fact, it spans a substantial part of the Sierras Pampeanas ranges, which attain heights above 6000 meters a.s.l. However, apart from a few small drainage networks in the east, the topography does not possess sufficient diversity in length scales as to contribute to a large complexity index.

Finally, inset D shows a region of intermediate values of *Q*, coinciding with the Puna plateau and comprising many high peaks, visible as bright dots. Complexity decreases westward, where topography becomes less intricate at the Atacama desert. Also eastward, beyond a zone with several small drainage systems, smaller complexity indexes unveil the connection with the ample low-*Q* region to the south. These examples illustrate a concrete use of quantifying image complexity, in this case, revealing nontrivial geometrical features of topographical traits.

## Methods and mathematical proofs

In this section, we first describe the two main procedures used to quantify complexity in a B/W digital image, namely, the measurement of the gray-level mean variance as a function of the scale, *V* (*S*), and the definition and computation of the complexity index *Q*. Then, we outline mathematical proofs for the connection between the maximum of *V* (*S*) and the typical scale of a periodic pattern, and for the algebraic dependence of *V* (*S*) for random and ordered images.

### Gray-level mean variance as a function of scale

For a given original B/W image of *N* × *M* pixels in size, we obtained different versions of the image with reduced resolution, using a standard resizing algorithm. In practice, we employed the ImageResize built-in function of Mathematica, choosing for resampling a nearest-neighbor symmetric interpolation kernel [28]. Each resolution was defined by a reduced image size of *N*′ × *M*′ pixels, with *N*′/*M*′ = *N*/*M* so that the aspect ratio was preserved. For the 360 × 540 images shown in Figs 1 to 4 (*N*/*M* = 2/3), we chose a series of 33 values of *N*′, given by the set {360, 328, 300, 272, 248, 224, 204, 188, 168, 156, 140, 128, 116, 108, 96, 88, 80, 72, 68, 64, 60, 56, 52, 48, 44, 40, 36, 28, 24, 20, 16, 12, 8}. For the 64 × 64 sub-images of the South-American topography image (*N*/*M* = 1), the values of *N*′ were {64, 56, 48, 40, 32, 24, 16, 8}.

At each resolution, we divided the *N*′ × *M*′ image into non-overlapping boxes of size *L* × *L*. To avoid cropping, *L* was chosen among the common divisors of *N*′ and *M*′ (effectively, as explained below, we always took *L* = 2). For each box *b*, we calculated the sample variance of the gray level *g*_*i*_ for the pixels *i* inside the box:

$$\begin{array}{c} V_{b}=\frac{1}{L^{2}-1}\sum_{i\;\text{in}\;b}{\left(g_{i}-{\bar{g}}_{b}\right)}^{2}, \end{array}$$ (1)

with

$$\begin{array}{c} {\bar{g}}_{b}=\frac{1}{L^{2}}\sum_{i\;\text{in}\;b}g_{i} \end{array}$$ (2)

the mean value of *g*_*i*_ over the box. The gray-level mean variance *V* was computed as the average of *V*_*b*_ over the *N*′*M*′/*L*^2^ boxes:

$$\begin{array}{c} V=\frac{L^{2}}{N^{\prime }M^{\prime }}\sum_{b}V_{b}. \end{array}$$ (3)

This value of *V* was assigned to a scale *S* such that the *L* × *L* box in the reduced image corresponded to an *S* × *S* box in the original image, namely,

$$\begin{array}{c} S=\frac{LN}{N^{\prime }}=\frac{LM}{M^{\prime }}. \end{array}$$ (4)

For the above quoted values of *N*′ and *L*, the scale *S* varied between *S*_min_ = 2 for *N*′ = 360 and *S*_max_ = 90 for *N*′ = 8.

As far as the boxes over which the gray-level variance is calculated are not too large as compared with the whole image, the choice of the size *L* is in principle arbitrary. However, we verified that –as may have been expected– the smaller the value of *L*, the sharper the definition of typical scales in the plot of *V* as a function of *S*. For instance, the maxima in *V* (*S*) for the images considered in Fig 1 were better defined for small *L*. For larger *L*, the maxima persisted, but became flatter. In view of this observation, we used *L* = 2 all along our study.

Note that the steps of resolution change and variance computation cannot be interchanged with each other, or aggregated into a single step where the variance is calculated over increasingly large boxes, without altering the result for *V* (*S*). In fact, the variance is a nonlinear function of the gray levels, while resolution reduction is, essentially, a linear average of grays. The separation of the two stages is crucial to the identification of typical scales with the values of *S* at which *V* (*S*) attains its maxima [24].

The chessboard-like images of Figs 1 and 2, as well as the random and ordered versions of Mona Lisa (Fig 3) were produced by ourselves. The other images are processed versions of copyright-free images downloaded from the public domain in the World-Wide Web or of photographs taken by ourselves. The original images were cropped, color-desaturated, and changed in brightness and color to suit the present purposes. The analyzed images are available as S1 File, in compressed format.

### Complexity index for B/W images

As discussed in the Results section, complexity can be quantified by the degree of flatness in the profile of the gray-scale mean variance as a function of the scale which, for each scale, is measured by the derivative *dV*/*dS*. We have seen that the values of both *V* and *S* can span wide intervals, which makes the log-log graphical representation used in Figs 1 to 4 more suitable to encompass the results of the computation of *V* (*S*). Accordingly, the quantification of the flatness of the variance as a function of the scale is better achieved using the logarithmic variables *v* = log *V* and *s* = log *S*, through the derivative *dv*/*ds* = (*S*/*V*)*dV*/*dS*. Note that *dv*/*ds*, which measures the slope of *V* (*S*) at each point in the log-log plot, does not depend on the selected logarithm base, as long as it is the same for *v* and *s*.

Taking into account that the slopes ±2 are close to the profiles associated with images of –arguably– minimal complexity (see Fig 2), the quantity $q\left(s\right)=1-\frac{1}{4}{\left(dv/ds\right)}^{2}$ can be proposed as a measure of the contribution of each scale to the complexity of the image under analysis. Namely, the flatter the variance profile at *s*, the smaller the derivative *dv*/*ds*, and the larger *q*(*s*). Conversely, the closer *dv*/*ds* to ±2, the smaller *q*(*s*). In principle, moreover, it would be possible that *q*(*s*) attains negative values, in the case where the variance profile becomes so steep that (*dv*/*ds*)^2^ > 4. In the following, we disregard these contributions of “negative complexity”. To aggregate the information provided by the function *q*(*s*) into a single complexity index, we define

$$\begin{array}{c} Q=\frac{1}{s_{\max}-s_{\text{min}}}\int_{s_{\text{min}}}^{s_{\text{max}}}[1-\frac{1}{4}{\left(\frac{dv}{ds}\right)}^{2}{]}_{+}ds, \end{array}$$ (5)

as the integral of *q*(*s*) over *s*, between the limits given by the minimal and maximal scales used in the analysis of the image in question, *s*_min_ and *s*_max_. Integration intervals where *q*(*s*) may be negative are excluded using the ramp function: [*x*]_+_ = *x* if *x* ≥ 0, and [*x*]_+_ = 0 otherwise. The prefactor (*s*_max_ − *s*_min_)^−1^ restricts the complexity index to the interval [0, 1] and, at the same time, makes *Q* independent of the logarithm base used to define *s*. The complexity index would reach its maximal value, *Q* = 1, for an image with equal variance at all scales, for which *V* (*S*) has a perfectly flat profile. At the other end, *Q* would be zero for profiles with slopes equal to or steeper than ±2 for all *S*.

Note that the index *Q* is invariant under a linear transformation of the gray level, $g_{i}^{\prime }=\alpha g_{i}+\beta$, with *α* and *β* constants. The additive constant *β*, in fact, is irrelevant to the calculation of the sample variance *V*_*b*_ in Eq (1) as it cancels in the difference between the gray level $g_{i}^{\prime }$ and its average ${\bar{g}}_{b}^{\prime }$. The prefactor *α*, in turn, affects the variance: $V_{b}^{\prime }=\alpha^{2}V_{b}$, and thus *V*′(*S*) = *α*^2^*V* (*S*). However, this prefactor disappears when we take logarithms and differentiate with respect to *s*:

$$\begin{array}{c} \frac{dv^{\prime }}{ds}=\frac{d}{ds}\text{log}\;V^{\prime }=\frac{d}{ds}\left(2\;\text{log}\;\alpha +\text{log}\;V\right)=\frac{d}{ds}\;\text{log}\;V=\frac{dv}{ds}. \end{array}$$ (6)

The above linear transformation amounts to change the image contrast and brightness [29]. Hence, leaving aside truncation effects due to the fact that the gray level is an integer number between 0 and 255, the complexity index does not depend on the degree of contrast and brightness of the image. This independence is further commented on in the Discussion section.

Naturally, as explained in the previous subsection, the procedure employed here to compute *V* (*S*) yields the variance for several discrete values of the scale only. The examples presented in Figs 1 to 4, moreover, clearly show that *V* can display moderate irregular fluctuations between neighboring values of *S*. In practice, thus, calculation of the integral that defines the complexity index *Q* in Eq (5) requires to conventionally introduce some kind of interpolation for the computed values of *V* (*S*). In the results reported in Table 1 we have used B-spline unclamped piecewise interpolations of maximal degree, plotted in the figures as curves. For the South-American topography sub-images, where the number of values of *S* was considerably smaller, we employed instead a third-degree polynomial interpolation.

### Mathematical model for the mean variance of a periodic pattern

In the following, by means of a simple one-dimensional model, we show that the mean variance of a pattern with a well-defined typical length exhibits a maximum at a scale which is directly related to that length. The model is inspired in a similar argument given in the framework of scale-space theory [24]. Let *g*(*x*) = *g*_0_ + *g*_1_ cos(2*πx*/λ) model the gray-level distribution along a continuous coordinate *x*. Clearly, *g*(*x*) represents a periodic pattern of wavelength λ –a kind of one-dimensional proxy of the chessboard-like images of Fig 1. For convenience in the calculations, we assume that the unit of measure on *x* is equivalent to one pixel.

In the context of this model, the procedure of resolution reduction applied in the main text –which is realized by resizing of the image, from *N* × *M* to *N*′ × *M*′– can be represented as an averaging of *g*(*x*) over a length *γ* = *N*/*N*′, plus a rescaling by a factor *γ* in the coordinate *x*. The new gray-level distribution is

$$\begin{array}{c} g_{\gamma }\left(x\right)=\int K_{\gamma }\left(y-\gamma x\right)g\left(y\right)\;dy, \end{array}$$ (7)

where *K*_*γ*_(*y*) is the averaging kernel. Using the Gaussian kernel *K*_*γ*_(*y*) = (2*πγ*^2^)^−1/2^ exp(−*y*^2^/2*γ*^2^), and neglecting boundary effects, we get *g*_*γ*_(*x*) = *g*_0_ + *g*_1_ exp(−2*π*^2^ *γ*^2^/λ^2^) cos(2*πγx*/λ).

Once *g*_*γ*_(*x*) has been obtained, we compute its variance over an interval of length *L* and average the variance over the coordinate *x*, getting the mean variance $V_{\gamma }=\pi^{2}\gamma^{2}L^{2}g_{1}^{2}\text{exp}\left(-4\pi^{2}\gamma^{2}/\lambda^{2}\right)/6\lambda^{2}$. To obtain this result, we have assumed that *L* ≪ λ, i.e. that the averaging length is small as compared with the typical length of the original pattern. Taking into account Eq (4), we have *S* = *γL*. Replacing *γ* = *S*/*L* in the above result for *V*_*γ*_, we get

$$\begin{array}{c} V\left(S\right)=\frac{\pi^{2}}{6}{\left(\frac{S}{\lambda }\right)}^{2}g_{1}^{2}\text{exp}(-\frac{4\pi^{2}S^{2}}{L^{2}\lambda^{2}}). \end{array}$$ (8)

This form of the mean variance as a function of the scale has a maximum at *S* = *L*λ/2*π*. The position of the maximum, therefore, is directly proportional to the typical length λ of the original pattern *g*(*x*).

It is interesting to mention that this calculation can be easily extended to the case where the analyzed signal is a linear combination of periodic functions, such as *g*(*x*) = *g*_0_ + *g*_1_ cos(2*πx*/λ_1_) + *g*_2_ cos(2*πx*/λ_2_) + ⋯ (although the algebra becomes considerably more tedious!). When the periods of the combined functions are mutually incommensurate, the signal is *quasiperiodic*. It can be proven that, if the length *L* is much shorter than λ_1_, λ_2_, … and the mean variance is computed over a sufficiently long interval over the variable *x*, much longer than λ_1_, λ_2_, …, contributions to *V* (*S*) coming from the products of two different terms in *g*(*x*) –which interfere with each other– will be negligible as compared with the contributions coming from the square of each single term –which reinforce themselves. As a consequence, *V* (*S*) will be approximately given by a sum of terms involving each individual periodic function. If the periods are sufficiently separated from each other, different contributions will be revealed as different maxima in *V* (*S*). The procedure in this case mimics harmonic analysis, not unlike a Fourier transform.

### Mean variance for random and ordered images

The algebraic relation between mean variance and scale for a random image, *V* (*S*) ∝ *S*^−2^, is a direct consequence of the fact that resizing the original image from *N* × *M* to *N*′ × *M*′ effectively conveys an average of the original gray level *g*_*i*_ over (*N*/*N*′)^2^ pixels. If the variance of *g*_*i*_ in the original random image is $\sigma_{g}^{2}$, averaging *g*_*i*_ over (*N*/*N*′)^2^ produces new random variables $g_{i}^{\prime }$ with a lower variance, $\sigma_{g^{\prime }}^{2}={\left(N/N^{\prime }\right)}^{-2}\sigma_{g}^{2}=L^{2}\sigma_{g}^{2}S^{-2}$; cf. Eq (4). This is an immediate byproduct of the well-known Central Limit Theorem. Due to the overall homogeneity of the random image, when the local variances, calculated over *L* × *L* boxes, are averaged to obtain the mean variance, the same relation holds: *V*_*g*′_ = *L*^2^*V*_*g*_*S*^−2^. Thus, for a given value of *L*, the mean variance decays proportionally to the inverse squared scale, *S*^−2^.

For an ordered image as in Fig 3, the main contribution to the gray-level variance comes from the variation of brightness along a single direction –in the case of the figure, the horizontal direction. Thus, the gray-level distribution is essentially one-dimensional, much as in the model considered in the preceding subsection. Suppose that, at a given resolution, the gray-level distribution along the direction of main variation is linear, so that $g_{i}^{\prime }$ varies at constant rate between its minimal and maximal values, $g_{\text{min}}^{\prime }$ and $g_{\text{max}}^{\prime }$. If the image size along that direction is *N*′, the gray-level profile at that resolution can be represented by the linear function

$$\begin{array}{c} g^{\prime }\left(x\right)=g_{\text{min}}^{\prime }+\frac{g_{\text{max}}^{\prime }-g_{\text{min}}^{\prime }}{N^{\prime }}x, \end{array}$$ (9)

with *x* varying from 0 to *N*′. The variance of *g*′(*x*) over a length *L* along the variable *x* is

$$\begin{array}{c} \sigma_{g^{\prime }}^{2}=\frac{L^{2}}{12}{\left(\frac{g_{\text{max}}^{\prime }-g_{\text{min}}^{\prime }}{N^{\prime }}\right)}^{2}\approx \frac{S^{2}}{12}{\left(\frac{g_{\text{max}}-g_{\text{min}}}{N}\right)}^{2}; \end{array}$$ (10)

cf. Eq (4). Note that $\sigma_{g^{\prime }}^{2}$ is independent of *x*, so that the mean variance is $V_{g^{\prime }}=\sigma_{g^{\prime }}^{2}$ and, therefore, *V*_*g*′_ ∝ *S*^2^. To obtain the above dependence of $\sigma_{g^{\prime }}^{2}$ on *S*, we have assumed that the extreme gray-level values are maintained by image resizing: $g_{\text{max},\text{min}}^{\prime }\approx g_{\text{max},\text{min}}$. This is expected to be the case, at least, for moderate resolution reductions of the ordered image. Moreover, since resolution reductions are performed keeping the aspect ratio constant, the proportionality $\sigma_{g^{\prime }}^{2}\propto S^{2}$ also holds if, in the direction of main brightness variation, the size of the image is *M*′ instead of *N*′.

For any given image –for instance, Mona Lisa– an ordered rearrangement of its pixels will generally not produce a linear gray-level profile as assumed above. However, on the average, the profile slope along the direction of main brightness variation will always be given by $\left(g_{\text{max}}^{\prime }-g_{\text{min}}^{\prime }\right)/N^{\prime }$ (or with *M*′ instead of *N*′). Therefore, by virtue of Eq (4), a sufficiently smooth profile will produce a mean variance approximately proportional to *S*^2^, as shown to happen for Mona Lisa in Fig 3.

## Discussion

We have proposed a complexity measure for black-and-white (B/W) digital images, on the basis of a quantification of the structural richness –as given by the diversity of typical length scales– in the depicted motifs. The results of this quantification are condensed in a single quantity, the complexity index, essentially defined as a distance to two extreme cases associated with minimal complexity: fully random and fully ordered distributions of gray levels. This definition of complexity as the distance to both order and disorder –which is aimed at capturing the balance between organization and diversity that we intuitively associate with complex systems [9, 10]– is implicit in other complexity measures. In out-of-equilibrium physical systems, for instance, complexity is quantified combining entropy, which increases as the system approaches equilibrium, with the degree of nonequilibrium, which grows in the opposite direction [16].

Our quantification of complexity conceives a B/W image as a distribution of gray levels over a rectangular two-dimensional domain. The complexity index is obtained through a series of mathematical operations applied to that distribution. These operations can in principle be applied to any distribution that admits a representation as a mathematical function over a suitably defined domain of arbitrary dimensionality. The complexity index could therefore be calculated for a broad variety of objects, within which the B/W images considered here are just a specific class, chosen for its convenience in the illustration of the method. The construction of a “complexity map” for South-American topography, although here performed on an image representation of the elevation data, is an example of such applications. It is worth pointing out that scale-space theory [23], on which we base the detection of typical scales in an image, has also been applied to time (auditory) signals [30] –namely, distributions defined over the one-dimensional temporal domain– and recently extended to deal with spatio-temporal (video, 2 + 1 dimensional) signals [31]. This opens wide perspectives for the use of the complexity index in connection with perception phenomena [20, 21]. In particular, whether the present complexity quantification bears a positive correlation with perceived complexity in images and other sensory signals should be decided on the basis of experimental research.

The complexity index *Q* defined here is but one way to integrate the information obtained from the detection of typical length scales. Our specific definition has mathematical properties that may be desirable in certain contexts, but that could be conveniently relaxed if necessary. In particular, recall that –as shown in the Methods section– *Q* is invariant under a linear transformation of gray levels, which amounts to changing the contrast and brightness of the image. In other words, up to rounding and saturation effects due to the discreteness of gray levels, our quantification assigns the same complexity to a given set of depicted motifs, independently of the degrees of contrast and brightness with which they are shown on the image. This independence sounds reasonable for an objective measure of structural intricacy, but it is not necessarily convenient from a perceptual viewpoint, where –particularly– contrast can affect visual complexity [20, 22, 32]. A dependence of the complexity index on contrast can be easily incorporated by weighting the contribution of each length scale with the gray-level mean variance, which is directly affected by contrast changes or, equivalently, using the variance instead of its logarithm in the definition of *Q*, Eq (5).

On the other hand, the effects of gray-level discreteness cannot be overlooked if contrast is decreased to such extreme that structural details in the image disappear because they collapse onto the same gray value. In this situation, increasingly larger portions of the image will have very similar gray levels, locally resembling the ordered image which, in our analysis, we have associated with one of the low-complexity extreme cases. This will translate into a decrease of *Q*, indicating a reduction of the image complexity. Note that, in the limit where the contrast has been reduced to such extent that the image becomes entirely monochromatic, the complexity index calculated as in Eq (5) is ill-defined. In fact, in this pathological case, the average variance is identically zero at all scales, and its logarithm –whose evaluation is necessary to compute *Q*– is undetermined.

Similar considerations regard the fact that the complexity index has been defined in such a way that it never exceeds the value *Q*_max_ = 1. This has been achieved by normalizing the contributions to complexity coming from different length scales by the ratio of maximal and minimal scales, which in turn depends on the image size. Such normalization has been useful in our comparison of images of the same size –the several rearrangements of Mona Lisa, and the sub-images of the South-American topography map– but, again, it might be not desirable in certain contexts. Is it reasonable, from a perceptual viewpoint, that image complexity has an upper bound? What happens with perceived visual complexity when images grow in size, in such a way that they can incorporate more and more details? To what extent can these details be cognitively grasped as elements of the same pictorial object? These questions call for further research, relating our proposed objective quantification of complexity to subjective assessments coming from empirical results.

## Supporting information

S1 File Compressed file containing the images analyzed in this work. (RAR)
